# Extreme strontium concentrations reveal specific biomineralization pathways in certain coccolithophores with implications for the Sr/Ca paleoproductivity proxy

**DOI:** 10.1371/journal.pone.0185655

**Published:** 2017-10-16

**Authors:** Michaël Hermoso, Benjamin Lefeuvre, Fabrice Minoletti, Marc de Rafélis

**Affiliations:** 1 Institut de Sciences de la Terre de Paris (UMR 7193), Sorbonne Universités, UPMC Univ Paris 06, CNRS, Paris, France; 2 Department of Earth Sciences, University of Oxford, Oxford, United Kingdom; 3 Géosciences Environnements Toulouse (UMR 5563 GET), Université de Toulouse III Paul Sabatier, CNRS, Toulouse, France; East China Normal University, CHINA

## Abstract

The formation of the coccolith biominerals by a group of marine algae (the Coccolithophores) offers fascinating research avenues both from the biological and geological sides. It is surprising how biomineralisation by a key phytoplanktonic group remains underconstrained, yet is influential on ocean alkalinity and responsible for the built up of our paleoclimatic archive over the last 200 Myrs. Here, we report two close relative coccolith taxa exhibiting substantial bioaccumulation of strontium: *Scyphosphaera* and *Pontosphaera* grown in the laboratory or retrieved from Pliocene sediments. This strontium enrichment relative to calcium is one order of magnitude greater than reported in other coccoliths of the orders Isochrysidales and Coccolithales, and extends well beyond established controls on Sr/Ca ratios by temperature and growth rate. We discuss this prominent vital effect in relation with possible specific uptake of strontium relative to calcium from the extracellular environment to the coccolith vesicle in coccolithophores excreting very large scale coccoliths. The report of Sr-rich biominerals challenges our current understanding of the cellular acquisition and intracellular trafficking of alkaline earth cations in phytoplanktonic calcifying eukaryotic algae. The presence of Sr-rich coccolith species in the geological record has to be quantitatively considered in future Sr/Ca-based palaeoceanographic reconstruction.

## Introduction

Besides mineralogy and taxonomy, a temperature and growth rate control have been identified as dictating the abundance of magnesium and strontium relative to calcium in biologically-precipitated carbonates [1–4]. This biogeochemical feature enables several proxies in paleoceanographic research [5–7]. Coccolithophore algae (Haptophyta) produce calcite biominerals, the coccoliths, intracellularly within a specialized compartment deriving from the Golgi body and called the coccolith vesicle. The presence of selective membranes around the cytoplasm and the coccolith vesicle renders the vital effect a complex phenomenon. Indeed, intracellular calcification in coccolithophores is a multi molecule-controlled process [8–11], and all the transport mechanisms of alkaline earth cations (Me^2+^) at play are not well understood. Calcium cations are abundant in seawater compared to the low cytosolic concentration that the cells have to maintain. Thus, the cytosol represents an electrochemical barrier between external seawater and the coccolith vesicle where Me^2+^ cations have to be pumped to in order to sustain calcification [12]. This Me^2+^ gradient represents a real challenge that is circumvented by various carrying strategies including Ca^2+^-permeable channels, Ca^2+^ ATPases and/or cation / Ca^2+^ exchangers [13–17]. These active transports likely represent a source of Sr enrichment in coccolith calcite compared to other biomineralizing organisms, such as the foraminifera that acquire Me^2+^ more directly, as by seawater vacuolization [1,11,18]. A biological control by cellular growth rate and the transport of Me^2+^ across the cell membrane has indeed been put forward to explain variations in Sr/Ca ratios in coccolith calcite: The faster pumping rate via transmembrane pumps, the higher coccolith Sr/Ca ratio [1]. From a mineralogical perspective, various controls on the partitioning coefficient of strontium in calcite (*D*_Sr_) by mineralogy, precipitation rate, surface topography of the crystal and pH exist, and entail a range of *D*_Sr_ from 0.02 to 0.65, including inorganic and biogenic calcite [19–23].

**Fig 1:**
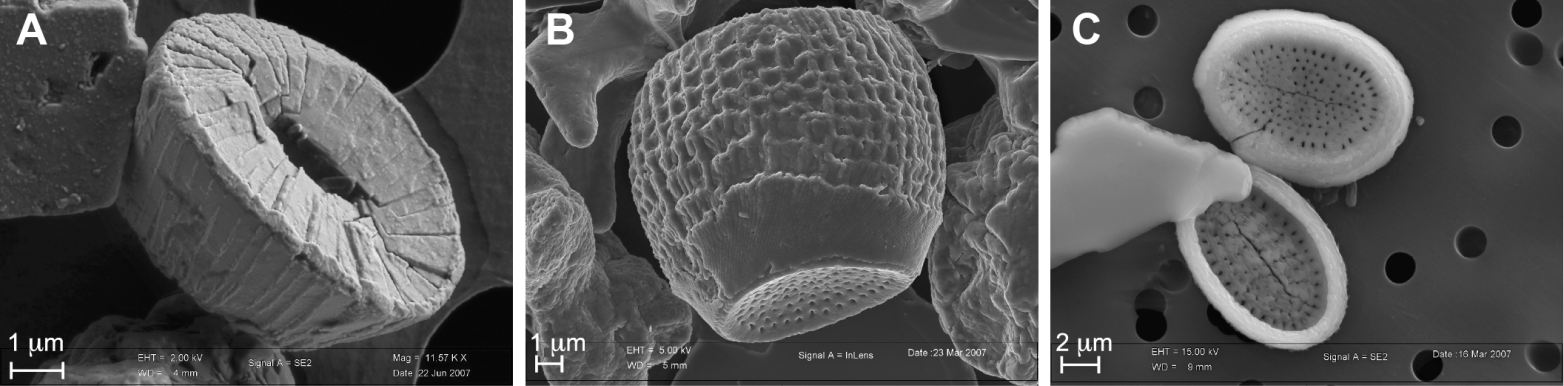
Scanning electron microscope images of the coccolithophore species examined in the present study. Panel (A) shows a close-up of *Crepidolithus crassus* from lower Toarcian sediments of the Paris Basin [25,31], and exhibiting a murolith shape. Panel b and Panel C show two coccoliths within the order Zygodiscale with distinct morphologies, but a rather similar ultrastructure: (B) *Scyphosphaera* (vase-shaped coccoliths or “lopadolith”) and (C) *Pontosphaera* (body coccoliths), both originating from Pliocene sediments of the Punta di Maiata section in Sicily [32,33].

**Fig 2:**
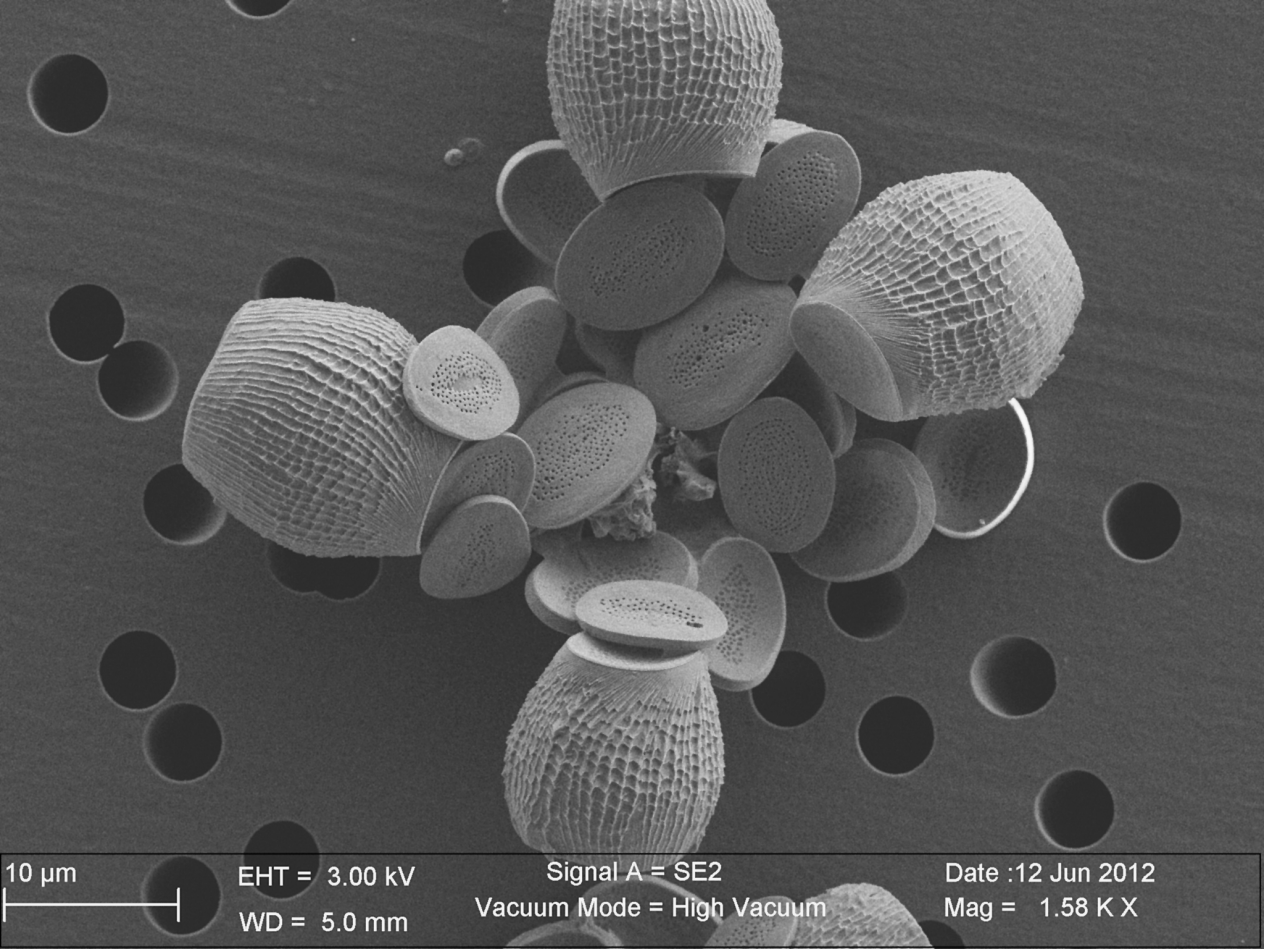
Collapsed coccosphere of *Scyphosphaera apsteinii* (strain RCC 1480) cultured in the present study. This coccolithophore cell was originally surrounded by two types of coccoliths during life, morphologically equivalent to the fossil specimens shown in Fig 1B and 1C. At the center of the image, a number of body coccoliths with a flat or bowl-shaped morphology are present, all exhibiting perforations of the central area. Originally, located in the equatorial plan of the coccosphere, four lopadoliths (vase-shaped coccoliths) were present. Although the morphologies and sizes of the two types of coccoliths differ, the ultrastructure of these two close relative taxa is rather similar (see text and ref [34,35] for further details).

Recent geological studies focusing on restricted time intervals of Earth history have challenged the view of rather uniform strontium concentrations among coccolith biominerals. In two distinct geological settings (Jurassic and Pliocene), coccolith-bearing sediments have been measured by wet chemistry with Sr/Ca ratios as high as 28 mmol/mol [24,25]. For reference, the typical concentration of strontium in pelagic carbonate is ~2 mmol/mol [26,27]. In both case studies, it was shown that not all the coccoliths forming the assemblage exhibited such elevated concentrations of strontium [24,25]. Rather, in each case, only one taxon displayed very high strontium contents: coccoliths of the genus *Crepidolithus* in the Toarcian (Fig 1A) and the coccoliths *Scyphosphaera* and *Pontosphaera* in the Pliocene (Fig 1B and 1C; Fig 2). At face value, the elemental range of strontium content relative to calcium represents an extraordinarily high degree of substitution of Ca^2+^ by Sr^2+^, more than can be accounted for by an environmental control by temperature on growth rate [1–4,11,28–30]. As an alternative, the presence of Sr-rich non-calcite phase within the biomineral may also explain such high measured concentrations.

Whilst *Crepidolithus* is extinct and has no identified descendant, *Scyphosphaera apsteinii* is still living and maintained *in vitro* in various culture collections. So, it has been possible to test for elevated strontium content of its coccoliths grown in controlled laboratory experiments. To do this, we performed Sr/Ca measurements on the coccoliths produced by *Scyphosphaera apsteinii* grown under varying seawater Sr/Ca ratios in the laboratory ranging from unsupplemented Sr concentrations (Sr/Ca = 8.8 mmol/mol) to Sr addition leading to a 16-fold-enrichement (~141 mmol/mol). The coccolithophore *Gephyrocapsa oceanica* was cultured in parallel in the same conditions and used as a control.

In the present study, we place these biogeochemical and elemental observations related to a notable Sr anomaly in discrete calcareous phytoplankton within a broader natural environment context, also investigating other key species, and comparing culture and calcareous nannofossil data. We discuss possible mechanistic constraints accounting for tremendous incorporation of Sr in coccoliths, a biogeochemical feature that can be ascribed to a vital effect in a key biological group with modern (biological) and paleoceanographic implications.

## Materials and methods

### Laboratory cultures of strains of calcifying coccolithophores

We cultured two species of coccolithophores in the laboratory in chemically modified media that were supplemented in strontium, hence with increasing Sr/Ca ratios. The two examined monoclonal coccolithophore strains comprise *Scyphosphaera apsteinii* strain RCC 1480 and *Gephyrocapsa oceanica* strain RCC 1314 (used as a control), both sourced from the Roscoff Culture Collection. The raw batch of seawater originated from the English Channel Sea and was measured with a Sr/Ca ratio of 8.83 mmol/mol, as determined in study [2], which analyzed the same batch. We added strontium chloride hexahydrate (Sigma–Aldrich; CAS Number 10025-70-4) to aliquots of the unmodified batch of seawater to to reach Sr_sw_ ×2; Sr_sw_ ×4; Sr_sw_ ×8; and Sr_sw_ ×16 enrichment relative to the unsupplemented (Sr_sw_ ×1) medium. Prior to inoculation, each medium was supplemented in trace metals (no Sr) and vitamins according to the *K*/2 recipe [36]. Medium pH was brought to 8.2 by addition of NaOH 0.2 N and then steri-filtered using a Millipore Stericup device. Cells were grown at 15°C in a Sanyo MLR 351 illuminated growth chamber under a daily 14h/10h light/dark cycle. The irradiance was ~150 μmol photons m^-2^ s^-1^. The cultures were conducted in 600 mL polycarbonate flasks with a 50 mL headspace and following a semi-continuous strategy by which the medium was refreshed every three days. Semi-continuous batches were implemented to ensure ambient Sr/Ca and other parameters (concentration of dissolved inorganic carbon and pH) remained constant over the course of the experiments [37,38]. In all bioassays, algal division rates were indistinguishable between Sr/Ca batches, with specific growth rates (μ) typically ~0.7 day^−1^ for *G*. *oceanica* and ~0.2 day^−1^ for *S*. *apsteinii*. Invariant growth rates with our Sr treatments shows the lack of influence of strontium concentration on algal growth within the range of Sr/Ca ratios applied in this study (~9 to 141 mmol/mol). Likewise, no differential ultrastructure or malformation features were noted under the SEM on specimens grown under different Sr concentrations. At harvest, the culture residues were collected by centrifugation and rinsed three times with neutralized deionized water. Subsequently, the culture residues were oxidized by H_2_O_2_ 20% neutralized by addition of 1N NaOH and left overnight to remove the organic phase, and then rinsed again as described above. The final culture residues were then gathered on polycarbonate membranes, air-dried and kept in a desiccating cabinet until further analysis.

### Sedimentary coccoliths from the Pliocene

The Pliocene portion of the Punta di Maiata section (~4 Ma) is exposed at the west coast of Sicily, and exhibits remarkable orbitally driven alternations of limestone and shale (marls) facies [32,39]. Bulk Sr/Ca measurements from sediments of this section indicate elevated ratios compared to averaged pelagic values [27,32]. In the present study, we measured coccoliths from sediments sampled from distinct alternating facies (limestone and marls) that originate from the bed belonging to cycles 39 and 43 in the work [33], at 46.11 m and 54.21 m in the section respectively. The sediments comprised coccoliths within the family Pontosphaeraceae including both the vase-like *Scyphosphaera* genus and the body coccoliths of the *Pontosphaera* genus. The relative abundance of these two taxa was around 1% of the assemblages of the sediments, whilst abundant coccolith taxa (> 10%) consisted in *Coccolithus*, *Calcidiscus*, *Helicosphaera* ssp..

### X-Ray diffraction analyses

Before we can meaningfully interpret measured Sr/Ca ratios from culture and sedimentary coccoliths, it is essential to constrain the mineralogy of the carbonate phase present. To this aim, we performed XRD analysis on a Bruker D2 Phaser device from gently crushed samples (cultures and sediments) prior to chemical analyses. The slides were prepared with non-oriented powders. The scans were performed from 3° to 75° at a scanning speed of 0.05° 2Θ/s, with counting steps of 1 second.

### Energy dispersive X-ray spectroscopy (EDS) measurements

Approximately 10 mg of culture or sediment samples was dispersed in neutralized deionized water in a 10 mL tube, and exposed to gentle ultrasonic treatment for 10–20 seconds. After sediment disaggregation, 0.5 mL of this suspension was pipetted out and pour onto a 2 μm polycarbonate membrane housed in a vacuum filter holder. The suspension was dried out after removing the liquid phase and left in a petri dish at room temperature for several hours until dry. A 1-cm^2^-large piece of this membrane was subsequently mounted on a stub using a carbon conductive adhesive, gold coated, and introduced in the instrument for imaging and spot chemical analysis. We used a Zeiss Ultra 55 Field Emission Scanning Electron Microscope fitted with field emission guns (Schottky-type) and a Bruker QUAD detector for X-Ray analysis. The working accelerating voltage (excitation energy) was 15 keV. Quantification of Sr and Ca was conducted from the L(α) peak of Sr at 1.8 keV La and from the K(α) ray of Ca at 3.69 keV, respectively. Raw results from EDS measurements provide atomic *wt*.% of Sr and Ca (among other elements). In this study, only the ratios between these two elements were used, as we refrained from attempting quantitative measurements due to the difficulty to quantify oxygen and carbon, and light elements, such as Mg. On the EDS spectra, the ray K(α) of Si and L(α) of Sr are very close (1.74 and 1.8 KeV, respectively) and overlap (S1 Fig). Although, the main peak in this energy region was centered on Sr, the Bruker Esprit 2 software resolved the Si/Sr overlap and confirmed very limited amount of Si in all calcite particles measured. In theory, the obtained Sr/Ca ratios can be directly converted into molar ratios accounting for the respective atomic masses of Ca and Sr (40.08 and 87.62 g mol^-1^, respectively) (Eq 1),

SrCa[mmol/mol]=1000×(Sr[wt.%]Ca[wt.%])×(40.0887.62) (1)

### EDS / ICP calibration of Sr/Ca ratios

We established a calibration on standard materials aiming at converting atomic *wt*.% into molar concentrations, and test the validity of Eq 1 in our study as Sr is known to be overestimated by the EDS technique. These natural samples included spar, nacre, aragonite, chiton, coral, *Halimeda* sp. (a green macroalgae with the ability to secrete calcium carbonate) and a specimen of oolith. Strontianite was also measured to assess the reliability of extrapolated Sr/Ca values above 11 mmol/mol. We performed about 12 spot chemical measurements on each standard material, randomly distributed on the surface of the mineral. For wet chemistry, powdered aliquots of approximately 1 g of the standards were digested by 1N acetic acid and chemically measured by ICP-AAS following the protocol described in ref [25]. The results are expressed as the means of all measurements from the EDS probe with those corresponding Sr/Ca ratios obtained by wet chemistry (S1 Table; S2 Fig). The following equation that relates Sr/Ca (*wt*.% / *wt*.%) to Sr/Ca (mmol / mol) can thus be proposed (Eq 2),

SrCa[mmolmol]=403.3×(Sr[wt.%]Ca[wt.%])−0.3 (2)

For reference, the theoretical slope of Eq 1 is 457.4 and a calibration line that would include the strontianite sample would have a rather similar slope of 436.7 (S2 Fig). It is worth noting that the analytical calibration is therefore relatively close to prediction (Eq 1). In details, EDS measurements are confirmed to slightly over-evaluate Sr concentrations, typically by 1.5 mmol/mol for Sr/Ca ratio of 0.05 *wt*.%. Further, the effect of the mineralogy (calcite vs aragonite) does not seem to affect EDS measurements. Retaining only the calcite standard would also lead to a similar slope of the regression line (422.9).

From these elemental molar ratios, we are then in position to calculate Sr partitioning coefficients (*D*_Sr_) of calcite (Eq 3), noting that these coefficients are only apparent as they report Sr partitioning between calcite and external environment rather than between calcite and the mineralizing fluid,

DSr=(SrCa)mineral(SrCa)medium (3)

## Results

### Partitioning coefficients of strontium in cultured coccoliths

**Fig 3:**
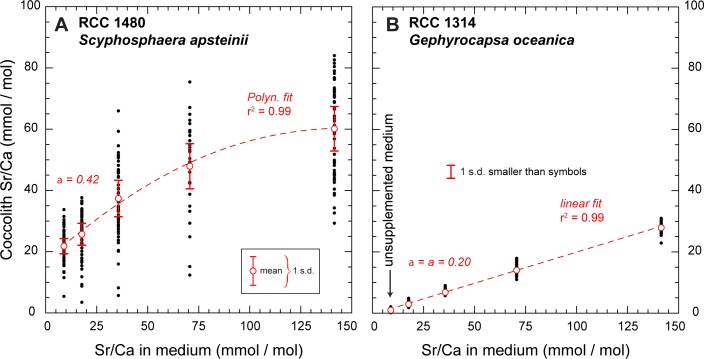
Cultured coccolith Sr/Ca ratios. Sr/Ca ratios measured from cultured *Scyphosphaera apsteinii* RCC 1480 (Panel A) and *Gephyrocapsa oceanica* RCC 1314 used as a control (Panel B). The *x* axis indicates discrete steps of medium enrichment in strontium ranging from 8.83 mmol/mol (unsupplemented medium) to 141.3 mmol/mol, representing 16-fold enrichment compared to natural seawater. In unsupplemented medium, Sr/Ca ratios are substantial in *S*. *apsteinii*, ~22.1 mmol/mmol on average and are ten times lower in *G*. *oceanica*, ~1.2 mmol/mol, in line with previous report on this latter species. We observe contrasting responses of the two examined species with increasing medium Sr/Ca ratios: a higher initial slope (α) in *S*. *apsteinii*, which becomes shallower at high ambient Sr/Ca ratios, and a linear relationship in *G*. *oceanica* across the entire range of medium Sr/Ca tested.

Field emission electron dispersive X-ray spectroscopy (FE-EDS) performed under the SEM enabled immediate detection of substantial amounts of strontium in all *Scyphosphaera apsteinii* coccoliths, as the main and characteristic L(α) peak of Sr at 1.8 keV was well expressed on the EDS spectra. From a mineralogical perspective, a few coccolithophore species within the Alisphaeraceae family have the ability to secrete aragonite liths [40] and may potentially explain Sr enrichment, as this phase is a Sr-rich carbonate phase with respect to calcite [41]. All cultured coccoliths have revealed to be pure calcite in this study, and no traces of aragonite have been detected. Likewise, no strontianite, sulfate or other accessory Sr-rich phases were detected. The systematic observation of elevated Sr content in *S*. *apsteinii* contrasted with measurements conducted on *G*. *oceanica* coccoliths for which the L(α) peak was much closer to the baseline. This preliminary qualitative assessment was subsequently confirmed and refined by semi-quantitative estimates of elemental of Sr/Ca ratios (see below). The K(α) peak at 14.16 keV was also evident for *Scyphosphaera* under increased excitation energy from 15 to 25 keV. These later analytical conditions enabled resolving the overlap between the Si and Sr peaks (S1 Fig). Converted into molar ratios thanks to a FE-EDS / ICP-AAS calibration (S1 Table; S2 Fig), the Sr/Ca ratios determined for *S*. *apsteinii* grown in the unmodified medium (Sr/Ca _medium_ = 8.8 mmol/mol) are comprised between 5.6 and 33.8 mmol/mol (Fig 3A). The mean value is 22.1 mmol/mol with one standard deviation (1 s.d.) of 5.2, which represents appreciable Sr/Ca ratios. The alga *Scyphosphaera apsteinii* produces two types of calcite mineralization with rather similar ultrastructure, but different morphologies–a feature termed dimorphism (Fig 2). The coccosphere is composed of plane or bowl-shaped body coccoliths with a narrow wall-like rim, whereas modified coccoliths with a strongly elevated rim leading to a vase-shaped morphology–*sometimes referred to as the lopadoliths* [34,42,43]–are implanted at the equatorial plan of the coccosphere. The body coccoliths synthetized by *Scyphosphaera* in culture look similar to *Pontosphaera* coccoliths found in the fossil record (Fig 1B and 1C). Identical mean Sr/Ca ratios were determined for the vase-shaped and body coccoliths. Likewise, there was no detectable difference in Sr/Ca ratios between spot analyses performed on the proximal and distal sides of the coccoliths. Rather, the deviation of data around the mean reflected an inter-specimen difference.

For *Gephyrocapsa oceanica*, the elemental ratios were significantly lower than in *Scyphosphaera apsteinii*, with a mean Sr/Ca value of 1.2 mmol/mol and a more restricted absolute dispersion of the data around the mean (1 s.d. = 0.5) (Fig 3B). We can consecutively determine the partitioning coefficients of strontium relative to calcium (*D*_Sr_) based on the ratios of these cations in medium and in calcite applying Eqs 2 and 3.

$$D_{Sr\;Scyphosphaera\;apsteinii}=2.5\pm 0.6\;(1\;s.d.)\;(n=56)$$

$$D_{Sr\;Gephyrocapsa\;oceanica}=0.14\pm 0.1\;(1\;s.d.)\;(n=55)$$

This latter figure for *G*. *oceanica* of the present study matches well previous reports from this species [2,7], and also from its close relative *E*. *huxleyi* measured by wet chemistry after acid digestion of calcite and measurements by mass spectrometry [1–3,7,11,44]. Previously reported *D*_Sr_ values from these studies ranged from 0.2 to 0.65. Besides the core experiments of the present study conducted on *S*. *apsteinii* and *G*. *oceanica*, a series of residues of the stock cultures sourced from the Roscoff Culture collection and maintained at the Oxford Biogeochemical Laboratories in unmodified medium with respect to natural seawater Sr/Ca ratio have been screened and no Sr enrichment was found in coccolith species comprising *Helicosphaera carteri* (RCC 1323), *Calcidiscus leptoporus* (RCC 1129), *Coccolithus pelagicus* (RCC 1202) and *Emiliania huxleyi* (RCC 1256). All the data fell within the range determined for *G*. *oceanica* and were in any case lower than 3 mmol/mol, therefore in line with published literature [7].

**Fig 4:**
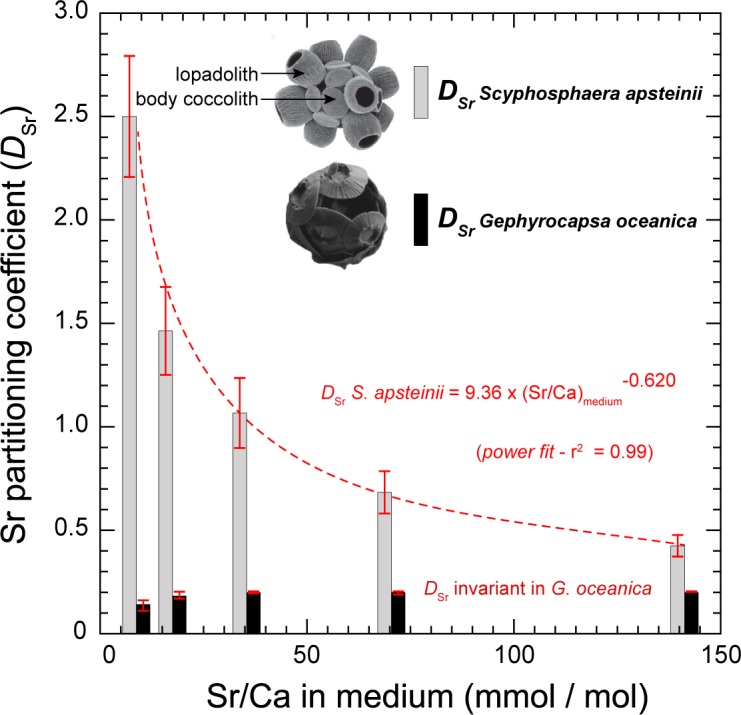
Calculated partitioning coefficients of strontium (*D*_Sr_) in coccolith calcite from the culture experiments. With the linear increase in Sr/Ca ratios in the culture media, *D*_Sr_ in *S*. *apsteinii* decreased exponentially according to a power function, leading to values of approximately 0.5 under the highest ambient Sr concentration. Meanwhile, the incorporation of Sr relative to Ca in *G*. *oceanica* coccoliths remained unaffected by our Sr treatments with constant *D*_Sr_ values around 0.26. The SEM images of coccosphere inset are from ref [43] for *S*. *apsteinii* and from the present study for *G*. *oceanica*.

We observe increased coccolith Sr/Ca ratios in both *S*. *apsteinii* and *G*. *oceanica* species with the supplementation of strontium to the culture media. However, the relative Sr enrichment in coccolith relative to each medium Sr/Ca condition differs between the two species (Figs 3 and 4). In *S*. *apsteinii*, the relationship linking coccolith and medium Sr/Ca ratios is not linear, but is best described by a second-order polynomial fit (r^2^ = 0.99) (Fig 3A). Nevertheless, the domain corresponding to Sr/Ca lower than 80 mmol/mol (hence excluding the Sr_sw_ ×16 data) can be assigned to a linear correlation with an initial slope (α) close to 0.4. At the highest medium Sr/Ca ratio, the data indicate that there is relatively less incorporation of strontium, hence lower *D*_Sr_ coefficients, compared to the unsupplemented conditions (Fig 4). In *G*. *oceanica*, we observe that medium and coccolith Sr/Ca covary with a linear fashion through the entire range of examined medium Sr/Ca (Fig 3B). The slope of the linear relationship between *G*. *oceanica* calcite and medium Sr/Ca ratios is 0.20. Thus, for *G*. *oceanica* a relatively constant *D*_Sr_ is observed with an average partitioning coefficient of 0.18 ± 0.03 (Fig 4).

### Strontium contents in sedimentary coccoliths

**Fig 5:**
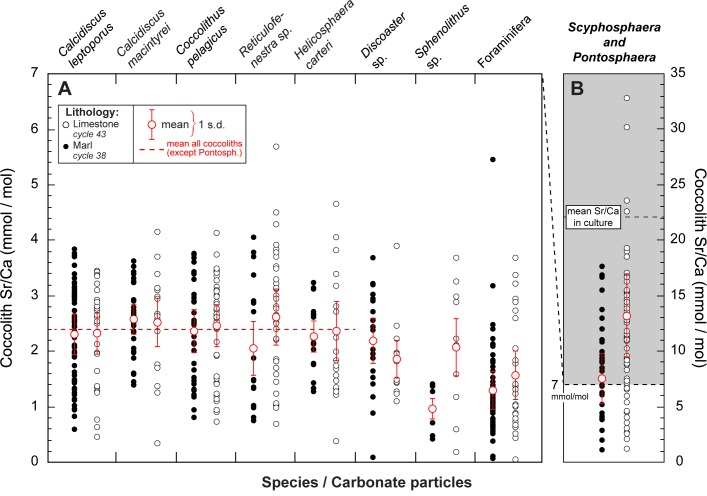
Pliocene coccolith Sr/Ca ratios. Results from the Pliocene case study (Neogene, Central Mediterranean Sea) showing relatively high Sr/Ca ratios in *Scyphosphaera* sp. and *Pontosphaera* sp. coccoliths compared to all other measured particles encountered in the two examined sediments (key to lithology is inset). Panel A shows Sr/Ca ratios for all abundant coccolith species, nannoliths and foraminifera debris. Panel B shows measurements conducted specifically on *Scyphosphaera* and *Pontosphaera* (please note the distinct scales used in the two panels). These latter coccolith taxa exhibit high Sr/Ca ratios, on the order of 10.4 mmol/mol with a large scattering of the individual analyses. Other coccoliths have an average Sr/Ca ratio around 2.4 mmol/mol (horizontal red dashed line), a value close to the elemental ratios reported for their modern counterparts.

All examined Pliocene *Scyphosphaera* sp. (vase-shaped) and the body coccoliths *Pontosphaera* sp. are confirmed to contain appreciable concentrations of strontium in sediments exposed in the Punta di Maiata section. The Sr/Ca ratios in *S*. *apsteinii* and *Pontosphaera* sp. were measured with an average value of 10.4 mmol/mol and a relatively large standard deviation of measurements of 6.0 (Fig 5). As found in the culture data, there is no differential Sr incorporation in the vase-shaped and body coccoliths. All other calcareous particles screened in the sediments exhibited Sr/Ca ratios below 5 mmol/mol. Other non-carbonate particles, such as quartz, feldspar or clays did not reveal a peak of Sr on the EDS spectra. Other coccoliths than *Scyphosphaera* sp. and *Pontosphaera* sp. in the assemblage did not show notable Sr enrichment compared to previously reported ratios for cultured and sedimentary coccoliths. Their average Sr/Ca ratio is 2.4 mmol/mol (Fig 5). These coccoliths mainly comprise *Coccolithus pelagicus*, *Calcidiscus* sp. and the reticulofenestrids (*Reticulofenestra* sp.). The nannoliths *Sphenolithus* sp. and *Discoaster* sp. bear also comparable amount of strontium to these coccoliths. Our study also confirms foraminiferal calcite as a less Sr-rich calcite than coccoliths [16,18]. In the Jurassic study case, the ratio in *Crepidolithus* sp. representing 5 to 15% of the calcareous nannofossil assemblages in the analyzed samples [45], was reported as high as 67 mmol/mol [25,46], whereas all other carbonate particles in the sediments were measured with significantly ratios (< 7 mmol/mol). There is, however, a difference in the Sr/Ca ratio of *S*. *apsteinii* between the two stratigraphic levels and rock facies. Meanwhile, no systematic Sr/Ca differences are noted in other coccolith species chemically analyzed from the two lithologies. We lastly note that Pliocene *S*. *apsteinii* coccoliths exhibit halved Sr/Ca ratios, on average, compared to the cultured specimens. Meaningful comparison between cultured and sedimentary coccoliths requires normalization of seawater Sr/Ca ratios in both cases that can be achieved through calculation of *D*_Sr_ values (Eq 3). The assumption of uniform seawater Sr/Ca ratio over the last 4 Ma seems to be backed up as normal coccoliths measured from Pliocene sediments have similar Sr/Ca ratios found in culture and recent settings [1–3,29,30]. From this observation emerges that cultured and sedimentary *Scyphosphaera* / *Pontosphaera* coccoliths exhibit statistically distinct enrichment in strontium relative to calcium, yet the mean *D*_Sr_ values of these muroliths in sediments is substantial, around 1.3 (Fig 5).

## Discussion

Our data unveil some species with Sr enrichment an order of magnitude greater compared to all previously measured coccoliths, such as *E*. *huxleyi*, *G*. *oceanica*, *C*. *pelagicus*, among others (Figs 3–5). The specificities of these coccolithophores and of their Sr-rich biominerals have to be compared to other coccoliths with “normal” *D*_Sr_ (~0.2) in order to discriminate between the two possible controls on this prominent vital effect. Indeed, appreciable amount of strontium can be incorporated into the coccoliths: *i*/ if strontium precipitation occurs from a mineralizing fluid with “normal” Sr/Ca ratio (hence very high *D*_Sr_), and *ii*/ if calcification is made from high Sr/Ca ratios in the coccolith vesicle (hence high *D*_Sr_ are only apparent).

### Coccolith ultrastructure, crystallography and *D*_Sr_ coefficients

Coccoliths are relatively complex biominerals formed of interlocking calcite crystals with crystallographic *c*-axis either vertically- or radially-oriented according to the coccolith plane. These monocrystals are termed *V*-units and *R*-units, respectively [47]. The differential growth pattern of the two sets of crystals is highly variable among taxa and controls the extinction pattern seen in cross-polarized light microscopy. A recently developed crystallochemical model of calcite growth relates the surface topography of the mineral and elemental ratios [22]. Calcification rate and patterns may thus influence the chemistry of calcite, in particular the degree of substitution of Ca^2+^ by other Me^2+^ cations [23]. Me^2+^ ions larger than Ca^2+^, such as Sr^2+^, are more readily incorporated into calcite units grown in the same direction as the *c*-axis, as they offer obtuse kink sites during mineral growth. Conversely, the incorporation of Me^2+^ smaller than Ca^2+^ is favored in calcite grown orthogonally to the *c-*axis. Therefore, strontium contents in coccoliths may be linked with growth pattern and ultrastructure, although this crystallochemical effect has proven to only slightly raises the Sr/Ca ratio of calcite with Δ*D*_Sr_ less than 0.2 [23]. The taxa *Scyphosphaera*, *Pontosphaera* and *Crepidolithus* have the notable commonalty to be muroliths. Their coccoliths are composed by elevated wall elements without well-developed shields (Figs 1 and 2). The Jurassic *Crepidolithus* muroliths are predominantly formed by *V*-units grown in the direction of the *c*-axis, and as such, this crystallographic pattern may favor the incorporation of strontium into calcite. In culture, *Scyphosphaera apsteinii* produces coccoliths mostly composed by *R*-units forming the inner rim and the central area, with *V*-units forming a narrow outer rim. In the case of the flat body coccoliths that can be related to *Pontosphaera* in the fossil record (Fig 1C), these *R*-units grow in the same direction as the *c*-axis and therefore may potentially bear high Sr contents. Conversely, the *R*-units of vase-like lopadoliths (Figs 1B and 2) grow orthogonally to the *c*-axis and should therefore exhibit relatively lower Sr contents–an observation that is not made in our study. Altogether, these crystallographic/chemical relationships do not appear to offer a straightforward explanation for Sr enrichment in coccoliths. Furthermore, the link between ultrastructure and strontium content of *Scyphosphaera* is also challenged by measurements from *Helicosphaera* sp. coccoliths, which have comparable ultrastructure and crystallographic orientations [35,42,48,49]. *Pontosphaera*, *Scyphosphaera* and *Helicosphaera* coccoliths are close relatives from a phylogenetic perspective, potentially offering biogeochemical commonalties, and perhaps the same Sr anomaly in all these taxa. However, cultured and sedimentary *Helicosphaera* sp. coccoliths exhibit low (“normal”) Sr/Ca ratios (Fig 5). Overall, a crystallographic control on Sr incorporation relative to calcium in coccoliths exists, but may account for only restricted variations in *D*_Sr_ values, on the order of 0.5 when taking the combined control by temperature and growth rate as a whole [1,23]. Such a relatively small change in *D*_Sr_ appears to be relatively minor and cannot be responsible for the exceptionally high ratios measured in *Scyphosphaera* and *Pontosphaera* (Fig 5). Thus, another mechanism that can potentially account for the order of magnitude difference in Sr/Ca ratios between coccoliths has to be sought.

### Decreased *D*_Sr_ in *Scyphosphaera* at extremely high medium Sr/Ca

Our Sr addition experiments showed contrasting responses in the accumulation of strontium in *Scyphosphaera apsteinii* and *Gephyrocapsa oceanica* (Figs 3 and 4). Under relatively low Sr ambient concentration, *S*. *apsteinii* exhibited greater enrichment in this element that may indicate active uptake (see later discussion). This hypothesis is mostly based on the observation of a steeper initial slope (α) in *S*. *apsteinii* compared to *G*. *oceanica* shown in Fig 3 (0.4 and 0.2, respectively). In contrast, the exponential decrease in *D*_Sr_ values of *S*. *apsteinii*, as Sr/Ca in the medium increased, reveals a limiting step in the incorporation of strontium into coccolith calcite (Fig 4). At the site of calcification, Me^2+^ cations compete with Ca^2+^ for calcite growth. The concentrations of Me^2+^, their physico-chemical properties (size, charge density, degree of solvation) and the surface structure of the mineral concurrently control the extent to which each Me^2+^ cation is being incrementally incorporated into calcite during growth. We suggest that at high Sr^2+^ concentration at the site of calcification, inter Sr^2+^–Sr^2+^ competition may take place and decrease net Sr^2+^–Ca^2+^ competition. This process offers a potential mechanism for substantial change in *D*_Sr_ in *S*. *apsteinii* in supplemented media (Fig 4). Meanwhile, *G*. *oceanica* is found to maintain a constant proportional Sr/Ca ratio between the extracellular and intracellular (coccolith vesicle) environments, as *D*_Sr_ coefficients did not evolve with varying ambient Sr/Ca concentrations. Overall, this explanation lends support to an enrichment of strontium specific to *S*. *apsteinii* in the coccolith vesicle upstream calcification.

### Seeking a biological control on coccolith Sr content

Uptake of calcium cations by coccolithophore cells is achieved via specific transmembrane proteins constituting Ca^2+^ channels, Ca^2+^ ATPase-dependent pumps and/or Ca^2+^/Me^2+^ (or Ca^2+^/H^+^) exchangers [9,10]. Vacuolization and fluid transport may represent complementary means to concentrate Me^2+^ to the coccolith vesicle [10,50], yet these processes are unlikely to discriminate Sr against Ca, as occurs for active pumping [1,11]. Intracellular biomineralization of the coccoliths is thus a highly regulated process, which requires continuous and massive pumping of Ca^2+^ (and dissolved inorganic carbon) to the coccolith vesicle where calcification takes place [10]. A conceptual model has been developed to link cellular growth rate and coccolith Sr/Ca ratio [1]. The rate of Me^2+^ transport through the cytoplasmic membrane has been suggested to covary with the uptake of strontium relative to calcium: The higher transport rate, the more Sr^2+^ enters the cell relative to Ca^2+^ [1]. This relationship forms the basis of the Sr/Ca-derived paleoproductivity proxy, and relies on the assumption that the rate of transmembrane pumping scales cellular growth taken as a whole. This hypothesis is backed up by the positive covariation between coccolith δ^13^C and Sr/Ca ratios in culture and the sedimentary record [1,30].

The nature of the transmembrane Me^2+^ transporters in coccolithophores remains underexplored [9,10,12]. Recent discovery of silicon transporters (SITs) in some coccolithophore species (including *S*. *apsteinii*) is a puzzling feature of the biomineralization toolbox employed by this biological group [51]. Transmembrane SITs allowing the uptake of silicic acid into the cytosol is a common and obligatory biochemical pathway used in diatoms. The nature of the SITs in *S*. *apsteinii* is reportedly distinct from those found in *Coccolithus pelagicus* and *Calcidiscus leptoporus*, potentially proving the specificity of this species regarding the assimilation of molecules from the external environment. Inside coccolithophore cells, the concentration of calcium to the coccolith vesicle is achieved by specific ionic transmembrane transporters deriving from the Golgi body. The transport of cations across the cytoplasm is a real problem since concentrations of Ca^2+^ are orders of magnitude lower in the cytoplasm than in seawater [10,52]. A detailed cellular description of the ultrastructure of *S*. *apsteinii* cells has revealed that the coccolith vesicle was closely associated with projections of the reticular body in this species [43]. Such an intracellular connection between the cell’s organelles may represent a means to supply sufficient Ca^2+^ to realize calcification of such large pieces of calcite, as in the case of *Scyphosphaera* and potentially *Crepidolithus*. As such, this Me^2+^ provision may represent a second and intracellular phase of Sr enrichment relative to Ca that follows the original step occurring across the cell’s membrane. Once concentrated in the coccolith vesicle, it is not clear if all cations are incorporated into coccoliths or if there is return flux from the coccolith vesicle to the cytosol forced by an electrochemical gradient. We suggest that the leakage of Ca^2+^ across Ca^2+^-permeable channels may represent a third step of Sr enrichment in the coccolith vesicle, leading to Sr/Ca ratios of the calcifying fluid much greater than in ambient seawater. This biological control may account for the relatively large variance of the Sr/Ca measurements conducted for the culture and sedimentary case studies. Further physiological research is required to better characterize the pathways employed by coccolithophore taxa, and specifically *S*. *apsteinii*, to accumulate alkaline earth metals necessary to biomineralization, and more broadly those used to maintain homeostasis with respect to intracellular calcium concentrations [10]. Our experimental data only provide access to ambient (seawater) and coccolith Sr/Ca ratios with no constraints on the intermediate steps during which the vital effect may be expressed. Yet, the differential (species specific) nature of the Sr anomaly reported here allows us to relate specific Me^2+^ trafficking across the cytosol with cellular ultrastructure and coccolith morphometry.

## Conclusions

The data of the present favor a hypothesis based on the concentration of Sr relative to Ca owing to the selectivity of the multiple membranes that separate the extracellular milieu from the site of calcification to explain extremely high Sr/Ca ratios in certain coccolith species. Our interpretations further imply that elevated *D*_Sr_ in *Scyphosphaera* calcite is only apparent, as that actual calcite precipitation process may incorporate “normal” amount of Sr relative to Ca with respect to the high ratios of the mineralizing fluid. A more extensive screening of extant and fossil species. Determining whether all the coccoliths within the Pontosphaeraceae family and related taxa in the geological record, based on the seminal work [49], exhibit high Sr content will be useful in the future to gain a more comprehensive picture of the Sr anomaly in coccolithophore calcite.

From a mineralogical perspective the presence of so large an amount of strontium within the trigonal lattice of calcite remains a puzzling feature, as the reported Sr/Ca ratios fall in a possible immiscibility gap among carbonate mineral species. Thus, this geochemical “anomaly” needs to be further explored by state-of-the-art crystallographic techniques, especially those able to detect Sr-rich nanophases associated to calcite crystals. Wherever strontium is hosted within the coccolith structure, the presence of substantial amount of strontium associated with laboratory-grown and fossil coccoliths has ramifications for the biomineralization toolbox in coccolithophores and for proxy data using the geochemistry of these abundant calcareous nannofossils that form a dominant fraction of pelagic sediments serving as a climatic archive.

## Supporting information

S1 Dataset Numerical dataset generated and used in the present study. (XLSX)

S1 Fig Example of EDS spectrum from spot chemical measurements on a cultured lopadolith specimens and showing the relative positions of Sr, Si and Ca and other elements under an excitation energy of 15 keV. (PDF)

S2 Fig Chemical calibration curves obtained from various biological and geological standards (S1 Table). The solid black line represents the raw conversion of Sr/Ca is *wt*.% to molar Sr/Ca ratios only accounting for the respective molar mass of calcium and strontium (Eq 1 in text). The thick dashed line is used to convert elemental *wt*.% to molar ratios in our study (Eq 2). The red dashed line corresponds to the linear regression obtained including strontianite (not shown on graph; see S1 Table for the numerical values).
(PDF)

S1 Table Standard materials used in the present study to establish a *wt*.% Sr/Ca to molar Sr/Ca ratios. (XLSX)
