# Supplementary material for: Extreme strontium concentrations reveal specific biomineralization pathways in certain coccolithophores with implications for the Sr/Ca paleoproductivity proxy
Source: PLoS One. 2017 Oct 16;12(10):e0185655. doi: 10.1371/journal.pone.0185655 (PMC5642888; doi:10.1371/journal.pone.0185655)
Supplement: S1 Fig — (PDF) [file pone.0185655.s002.pdf]

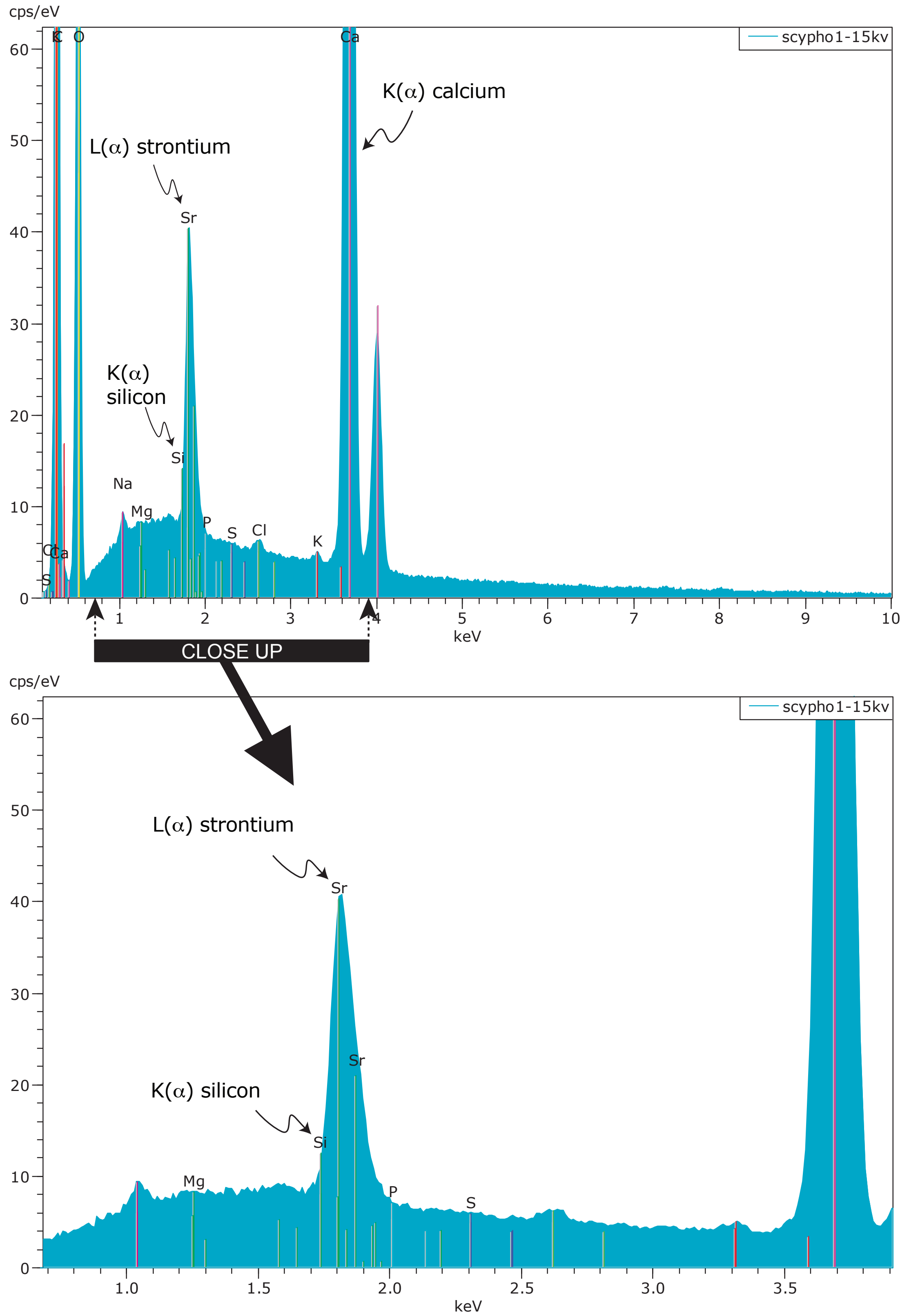

S1 Fig. Example of EDS spectrum from spot chemical measurements on a cultured lopadolith specimens and showing the relative positions of Sr, Si and Ca and other elements under an excitation energy of 15 keV.
