# Supplementary material for: Extreme strontium concentrations reveal specific biomineralization pathways in certain coccolithophores with implications for the Sr/Ca paleoproductivity proxy
Source: PLoS One. 2017 Oct 16;12(10):e0185655. doi: 10.1371/journal.pone.0185655 (PMC5642888; doi:10.1371/journal.pone.0185655)
Supplement: S2 Fig — The solid black line represents the raw conversion of Sr/Ca is wt.% to molar Sr/Ca ratios only accounting for the respective molar mass of calcium and strontium (Eq 1 in text). The thick dashed line is used to convert elemental wt.% to molar ratios in our study (Eq 2). The red dashed line corresponds to the linear regression obtained including strontianite (not shown on graph; see S1 Table for the numerical values). (PDF) [file pone.0185655.s003.pdf]

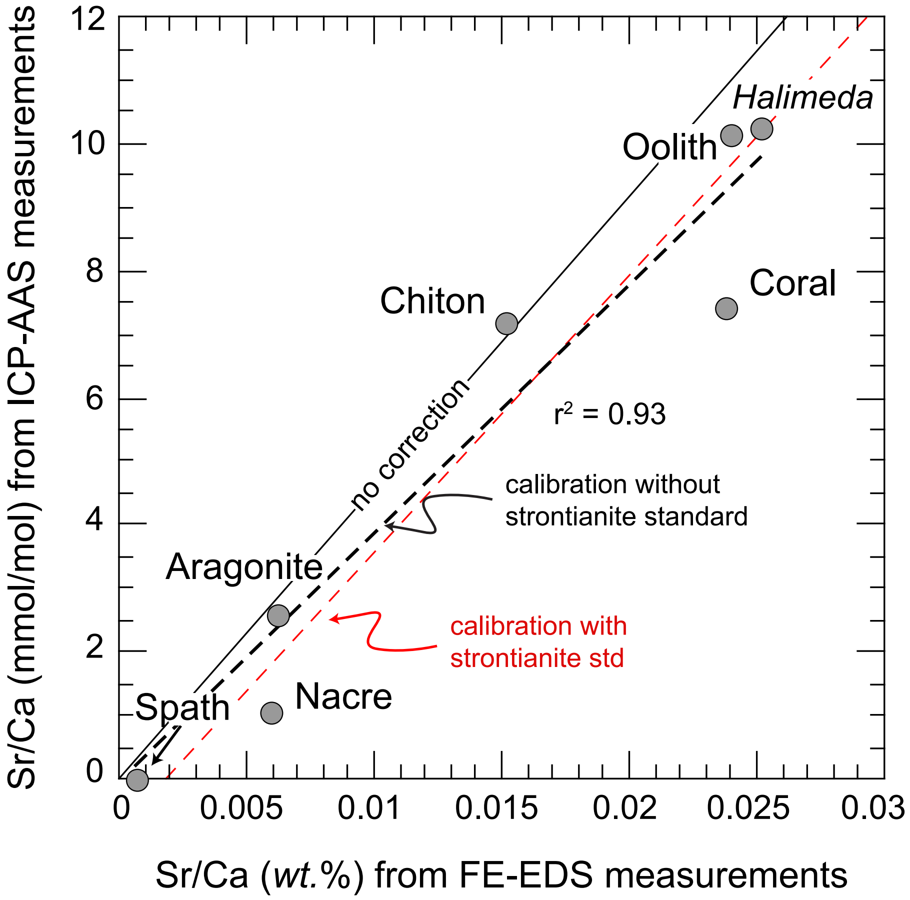

S2 Fig. Chemical calibration curves obtained from various biological and geological standards (S1 Table). The solid black line represents the raw conversion of Sr/Ca is wt.% to molar Sr/Ca ratios only accounting for the respective molar mass of calcium and strontium (Eq. 1 in text). The thick dashed line is used to convert elemental wt.% to molar ratios in our study (Eq. 2). The red dashed line corresponds to the linear regression obtained including strontianite (not shown on graph; see Table S1 for the numerical values).
